# Links between air pollution and COVID-19 in England^☆^

**DOI:** 10.1016/j.envpol.2020.115859

**Published:** 2021-01-01

**Authors:** Marco Travaglio, Yizhou Yu, Rebeka Popovic, Liza Selley, Nuno Santos Leal, Luis Miguel Martins

**Affiliations:** MRC Toxicology Unit, University of Cambridge, UK

**Keywords:** SARS-CoV-2, COVID-19, Air pollution, Nitrogen oxides, Ozone, PM_2.5_, PM_10_, Mortality

## Abstract

In December 2019, a novel disease, coronavirus disease 19 (COVID-19), emerged in Wuhan, People’s Republic of China. COVID-19 is caused by a novel coronavirus (SARS-CoV-2) presumed to have jumped species from another mammal to humans. This virus has caused a rapidly spreading global pandemic. To date, over 300,000 cases of COVID-19 have been reported in England and over 40,000 patients have died. While progress has been achieved in managing this disease, the factors in addition to age that affect the severity and mortality of COVID-19 have not been clearly identified. Recent studies of COVID-19 in several countries identified links between air pollution and death rates. Here, we explored potential links between major fossil fuel-related air pollutants and SARS-CoV-2 mortality in England. We compared current SARS-CoV-2 cases and deaths from public databases to both regional and subregional air pollution data monitored at multiple sites across England. After controlling for population density, age and median income, we show positive relationships between air pollutant concentrations, particularly nitrogen oxides, and COVID-19 mortality and infectivity. Using detailed UK Biobank data, we further show that PM_2.5_ was a major contributor to COVID-19 cases in England, as an increase of 1 m^3^ in the long-term average of PM_2.5_ was associated with a 12% increase in COVID-19 cases. The relationship between air pollution and COVID-19 withstands variations in the temporal scale of assessments (single-year vs 5-year average) and remains significant after adjusting for socioeconomic, demographic and health-related variables. We conclude that a small increase in air pollution leads to a large increase in the COVID-19 infectivity and mortality rate in England. This study provides a framework to guide both health and emissions policies in countries affected by this pandemic.

## Highlights

• Regional levels of NO_2_, NO and O_3_ correlate with number of COVID-19 deaths.
• Sub-regional NO_x_ levels are associated with increased COVID-19 deaths and cases.
• Levels of PM and NO_x_ map increased number of COVID-19 infections in the UK Biobank.

## Graphical abstract

**Figure:**
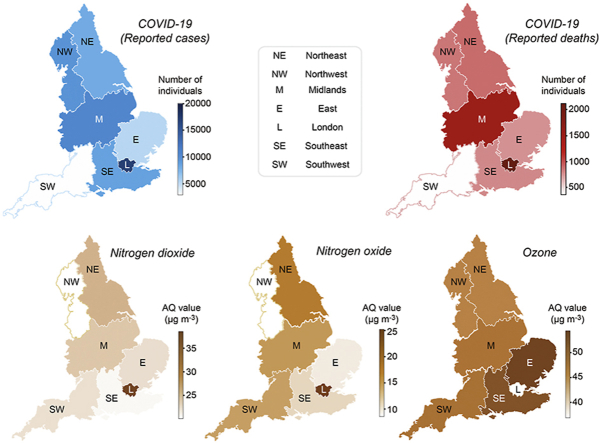

## Abbreviations

AIC: Akaike Information Criterion
AQ: Air quality
BEIS: Business, Energy and Industrial Strategy
CI: Confidence intervals
CoV: Coronavirus
COVID-19: Coronavirus disease 19
DEFRA: Department for Environment, Food and Rural Affairs
DfT: Department for Transport
GHGI: Greenhouse Gas Inventory
HGV: Heavy goods vehicle
LGV: Light goods vehicle
MPRN: Meter point reference numbers
NAEI: National Atmospheric Emissions Inventory
NHS: National Health Service
PCA: Principal component analysis
PM: Particulate matter
PM2.5: Particulate matter with an aerodynamic diameter <2.5 μm
PM10: Particulate matter with an aerodynamic diameter <10.0 μm
PHE: Public Health England
SARS: Severe acute respiratory syndrome
SARS-CoV-2: Severe acute respiratory syndrome coronavirus 2
WHO: World Health Organization

## 1 Introduction

In December 2019, a high number of pneumonia cases with an unknown aetiology were detected in Wuhan, China. A molecular analysis of samples from affected patients revealed that their symptoms were caused by an infection with a novel coronavirus, later named severe acute respiratory syndrome (SARS) coronavirus (CoV) 2 (SARS-CoV-2), the pathogenic agent of coronavirus disease 19 (COVID-19) (Zhu et al., 2020a). Within five months, this disease had affected more than 210 countries and became a global pandemic, causing devastating consequences to public health (Wang et al., 2020a). Coronaviruses are a genus of enveloped, non-segmented, positive-sense RNA viruses belonging to the family Coronaviridae and classified within the Nidovirales order (Yi et al., 2020). Historically, illnesses caused by coronaviruses have ranged in severity, with some, including human coronaviruses-229E and -OC43, causing common cold symptoms, but SARS-CoV and Middle East respiratory syndrome coronavirus have initiated outbreaks of life-threatening pneumonia (Yi et al., 2020). While the initial symptoms of COVID-19 include fever with or without respiratory syndrome, a crescendo of pulmonary abnormalities may subsequently develop in patients (Huang et al., 2020). According to recent studies, most patients present with only a mild illness, but approximately 25% of hospital-admitted patients require intensive care because of viral pneumonia with respiratory complications (Wang et al., 2020a).

While extensive research into the pathogenesis of COVID-19 suggests that the severe disease likely stems from an excessive inflammatory response (Cao, 2020), the exact predisposing factors contributing to increased clinical severity and death in patients remain unclear. Individuals over the age of 60 years or with underlying health conditions, including cardiovascular and chronic respiratory diseases, diabetes, and cancer, are at the highest risk of increased clinical severity and death (Verity et al., 2020). Since several studies have shown that chronic exposure to air pollution enhances both respiratory and cardiovascular toxicity (Faustini et al., 2014), it has been hypothesised that air pollution may also contribute to COVID-19 severity (Brandt et al., 2020; Conticini et al., 2020). Early reports have shown that the geographical patterns of COVID-19 transmission and mortality among countries, and even among regions of single countries, closely align with local levels of air pollutants (Conticini et al., 2020). For example, increased contagiousness and COVID-19-related mortality in northern Italian regions, including Lombardia, Veneto and Emilia Romagna, have been correlated with high levels of air pollutants in these regions (Conticini et al., 2020). This hypothesis has become increasingly popular because despite the progress in characterising the clinical features of patients with COVID-19, details regarding the risk factors for clinically ill patients remain elusive.

A recent analysis by the Lancet Commission indicated that air pollution is responsible for 16% of global deaths, making it the primary cause of preventable premature death worldwide (Landrigan et al., 2018). Although the recent implementation of emergency lockdown measures has contributed to a considerable improvement in air quality around the world (Bherwani et al., 2020; Gautam, 2020a, (Gautam, 2020b); Muhammad et al., 2020), the levels of most air contaminants remain considerably higher than the values recommended by the WHO in several countries (He et al., 2020; Wang et al., 2020b). The rapid expansion of anthropogenic activities such as transportation, industrial processes and mining caused a widespread increase in many harmful pollutants that pose a major risk to human health (Sharma et al., 2020). For instance, prolonged exposure to common road transport pollutants, including nitrogen oxides and ground-level ozone, can induce oxidative stress and inflammation within the airways, inducing and significantly exacerbating health conditions such as asthma, chronic obstructive pulmonary disease, cardiovascular diseases and diabetes (Guarnieri and Balmes, 2014; Strak et al., 2017). These conditions have been shown to overlap with pathological features of COVID-19 critical illness, reinforcing the hypothesis of a dichotomy between air pollution and COVID-19 (Conticini et al., 2020; Wu et al., 2020). Furthermore, airborne particulate matter (PM) was recently shown to increase the viability of SARS-CoV-2, suggesting that direct microbial pathogenic transmission occurs through the air and the opportunity for infection is increased in highly polluted areas (Setti et al., 2020). Therefore, air pollution has been suggested to contribute to COVID-19 severity, either directly, by compromising the lungs’ immune response to the infection, or indirectly, by exacerbating underlying respiratory or cardiovascular diseases (Brandt et al., 2020; Conticini et al., 2020; Dutheil et al., 2020). However, most studies have failed to account for multiple confounding factors (Conticini et al., 2020; Ogen, 2020) while others have focused on relatively large geographical regions (Cole et al., 2020; Liang et al., 2020; Wu et al., 2020). A convincing link between COVID-19 and air pollution can only be established by combining data from available ambient sensors with individual-level information, where possible, to reduce uncertainty in exposure estimates based on ambient monitoring data.

Here, we aimed to explore the relationship between air pollution exposure and COVID-19 mortality and infectivity in England, at the population- and individual-level. In the UK, adverse air quality causes approximately 30,000 premature deaths a year, and the concentration of most air pollutants is predicted to exceed limits set by European Union (EU) legislation beyond 2030 (UK Goverment, 2019; Pannullo et al., 2017). For instance, data collected in 2018 shows that in England specifically, ambient nitrogen oxides concentrations exceed these limits in 89% of designated air quality assessment zones (Affairs, 2019). In addition, England experienced the highest excess all-cause mortality rate in Europe in the first five months of 2020 compared with 2015–19, making it one of the world’s most affected countries by the COVID-19 pandemic, according to recent data (Raleigh, 2020). These observations indicate that England provides a unique setting in which to examine the link between air pollution and COVID-19.

In this study, we first investigated potential links between regional and subregional variations in air pollution and population-level COVID-19-related deaths and cases in England by employing coarse and fine resolution methods. Next, we addressed these associations between air pollutants and the risk of COVID-19 infection at the individual scale by analysing UK Biobank data obtained from a cohort of 1464 subjects. Combining individual-level data on COVID-19 with high-resolution air pollution data, we show a clear link between long-term exposure to air pollution and COVID-19 in England. There are important, practical implications from this research. The identification of key modifiable environmental factors may contribute to mitigating the risk of COVID-19 and minimise the impact of future pandemics. Moreover, increased knowledge about the link between air pollution and COVID-19 may be beneficial worldwide by informing public health measures and disease management strategies in clinical practice.

## 2 Methods

### 2.1 Data sources for COVID-19 deaths and cases

Our study utilised regional-level, subregional-level and individual-level information to estimate the relationship between air pollution and COVID-19 in England. For our initial regional analysis, the number of patients infected with SARS-CoV-2 in England was obtained from Public Health England (PHE) and analysed according to the following statistical regions: London, Midlands, Northwest, Northeast and Yorkshire, Southeast, East, and Southwest England. Region-level data on the cumulative number of SARS-CoV-2-related deaths in England was retrieved from the National Health Service (NHS) (Table 1). This source provides one of the most comprehensive region-specific records of COVID-19-related deaths in England. The daily death summary included the number of deaths of patients who died in hospitals in England and had tested positive for SARS-CoV-2 at the time of death. While this online repository is updated daily, figures are subject to change due to a *post-mortem* confirmation of the diagnosis. Local authority-level data on the cumulative number of COVID-19 deaths in England was provided by the Office for National Statistics (ONS) (Table 1). This repository includes deaths of patients who died in care homes or other places outside hospitals. All deaths are recorded as the date of death rather than the day on which the death was announced.

**Table 1:** Summary of data sources.

Data type	Source	Download date	Measuring units
COVID-19 cases	Public Health England (https://coronavirus.data.gov.uk/#region)	April 9, 2020	Lab-confirmed cases per region up to and including April 8, 2020
COVID-19 deaths (regional)	National Health System (https://www.england.nhs.uk/statistics/statistical-%20work-areas/covid-19-daily-deaths/)	April 9, 2020	Cumulative death counts per region up to and including April 8, 2020
COVID-19 deaths (subregional)	Office for National Statistics (https://www.ons.gov.uk/peoplepopulationandcommunity/birthsdeathsandmarriages/deaths/bulletins/deathsregisteredweeklyinenglandandwalesprovisional/weekending1may2020/)	April 28, 2020	Cumulative death counts per local authority
COVID-19 cases (subregional)	Public Health England (https://coronavirus.data.gov.uk/#LA)	May 15, 2020	Cumulative cases counts per local authority
Nitrogen dioxide, nitrogen oxide and ozone concentrations	European Environmental Agency (EEA)(https://www.eea.europa.eu/data-and-maps/data/aqereporting-8)	April 7, 2020	AQ values (μg/m^3^)
Population data, mean annual earnings and median age	Office for National Statistics (https://www.ons.gov.uk)	April 17, 2020	Regional and subregional population density in England (person/km^2^). Age in years. Annual earnings in GBP.
Air quality data (Pollution Climate Mapping)	UK Air information resources (https://uk-air.defra.gov.uk/data/pcm-data)	May 2, 2020	AQ values (μg/m^3^), except for ozone: days in which the daily max 8-hr concentration is greater than 120 μg/m^3^
National emission totals	DEFRA(https://webarchive.nationalarchives.gov.uk/20200303104044/https://www.gov.uk/government/statistics/emissions-of-air-pollutants)	May 5, 2020	National emission totals by sector expressed in thousands of tonnes of oil equivalent (kToE)

This table summarizes publicly available data sources used for the analysis.

For our regional analysis, the cumulative number of COVID-19 cases and deaths was gathered from PHE (Table 1), and these data include over 61,613 lab-confirmed cases and 7248 deaths reported between February 1 and April 8, 2020. Local authority-level models included COVID-19 data reported in England between February and April 2020, which was approximately a month after England was placed on lockdown. COVID-19 data used for this analysis were obtained from the ONS and includes a total of 32,903 deaths and 103,409 lab-confirmed cases. As the cumulative number of COVID-19 cases in England were reported according to different time scales, COVID-19 cases used for this analysis include lab-confirmed cases reported up to and including April 26 whereas COVID-19 deaths include COVID-19-related deaths registered in England up to and including April 31. The first death involving COVID-19 in England occurred on March 2, 2020. Finally, for the individual-level analysis, we obtained COVID-19 data from the UK Biobank, where 1464 participants were tested for COVID-19 in England by April 26, 2020. Out of the total participants, 17 individuals were excluded from the final analysis due to incomplete data.

### 2.2 Data sources for air pollution levels

**Fig. 1:**
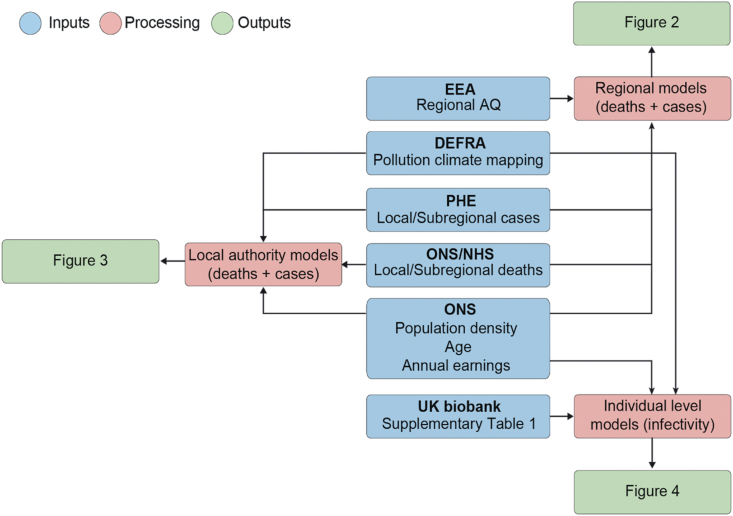
Analysis workflow.

**Fig. 2:**
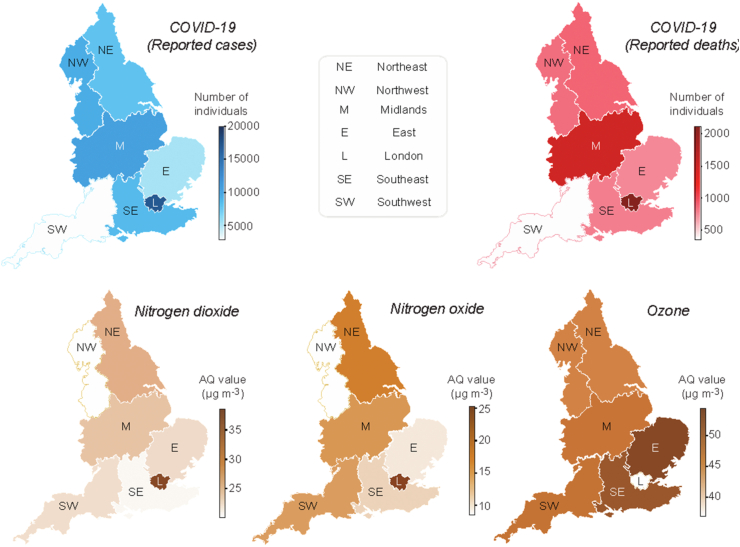
Regional heatmaps of COVID-19 and pollutants.

Air pollution data were obtained from two sources. For the initial region-level analysis, we collected annual aggregated air quality (AQ) values determined by the European Environmental Agency based on direct observations obtained from multiple monitoring stations located across England. Due to incomplete or obsolete observations for several pollutants, we restricted our regional analysis to three major air pollutants, namely, nitrogen dioxide, nitrogen oxide and ozone, across the prespecified regions (Fig. 2). Nitrogen dioxide, nitrogen oxide and ozone AQ values are reported in μg/m^3^ and represent the annual average of daily measurements for each air pollutant from 2018 to 2019 in each specified region. Air pollution data for the regional analysis could not be temporally averaged across multiple years because of the large inconsistencies in the data in the years prior to 2018. No data were available for the year 2019 at the time of writing. The identification of each monitoring station was matched to each available city by accessing the Department for Environment, Food and Rural Affairs (DEFRA) website (Fig. 1). This website contains a resource called the DEFRA’s Air Quality Spatial Object Register, which allows users to view and retrieve information on the air quality-related spatial and non-spatial data objects from the UK’s Air Quality e-Reporting data holdings. The annual average values of daily measurements for each pollutant in each monitoring area were analysed to determine the effects of toxin exposure on the number of SARS-CoV-2 cases and deaths across England (Fig. 1).

For the analysis at the level of local authorities, we used the Pollution Climate Mapping (PCM) data from the UK Air Information Resources (Table 1). This repository contains information from hundreds of air quality stations located across England for multiple pollutant molecules (ozone, nitrogen oxides, PM_2.5_, PM_10_). All data are shown as either annual average values of daily measurements for the year 2018 (single-year model) or temporally averaged levels for the years 2014–2018 (multi-year model) to capture the multi-year trajectory of historical change in air pollution in England. Air pollutant levels are reported in μg/m^3^, except for ozone, whose metric is the number of days on which the daily max 8-hr concentration is greater than 120 μg/m^3^. A detailed quality report regarding this data is available at the following website: https://uk-air.defra.gov.uk/assets/documents/reports/cat09/1903201606_AQ0650_2017_MAAQ_technical_report.pdf. We obtained the longitude and latitude of each local authority using OpenCage Geocoder (https://opencagedata.com/). The air pollutant levels for each authority was approximated by averaging 10 values nearest the centre of authority. This area covers approximately 12 km^2^. Detailed descriptions of the methodology and analysis workflow are available in our GitHub repository. For the UK Biobank data, we matched the location coordinate each participant reported to their nearest modelled value. The level of each pollutant was measured less than 2 km away from the self-reported address.

### 2.3 UK biobank data sources

We used data from the UK Biobank under application #60124. Details regarding the geographical regions, recruitment processes, and other characteristics have been previously described (Sudlow et al., 2015),^14^, and are found on ukbiobank.co.uk. The UK Biobank has received ethical approval from the North West – Haydock Research Ethics Committee, 11/NW/0382 to gather data from participants. A detailed list of the variables analysed in the present study is presented in Supplementary Table 1 (https://m1gus.github.io/AirPollutionCOVID19/). Notably, we defined hypertension using the criteria of a diastolic blood pressure ≥90 mmHg OR systolic blood pressure ≥140 mmHg. We assigned an average of annual pollutant concentrations from the PCM data to each study subject, on the basis of a six-digit postcode. Individual-level data were collected from the UK Biobank on April 26, 2020. This dataset contained information on individuals that tested positive for COVID-19. No COVID-19 test data were available for UKB assessment centers in Scotland and Wales, thus data from these centers were not included.

### 2.4 Regional heatmaps

Heatmap legends were generated using GraphPad Prism 8 (www.graphpad.com), and regions are labelled with the mapped colour values.

### 2.5 Statistical analysis

In our regional exploratory analysis, we fitted generalised linear models to our data using COVID-19 deaths and cases as the outcomes and nitrogen oxide, nitrogen dioxide and ozone as the exposures of interest, adding the corresponding population density values as a confounding variable. Population density (person/km^2^) data correspond to subnational mid-year population estimates of the resident population in England and excludes visitors or short-term immigrants (<12 months). We modelled both the number of cases and deaths using negative binomial regression analyses since the response variables are overdispersed count data. We used the same model type for our subregional analysis, adding mean annual earnings and median age as confounding factors.

For the UK Biobank models, we fitted a binomial regression model because the response variable, COVID-positive or -negative, is defined as either 0 or 1.

Methods for assessing the fit of the model included residual analyses, diagnostic tests, and information criterion fit statistics. The goodness of fit of each regression model was determined using the log-likelihood and Akaike Information Criterion (AIC) statistics.

For all models, we calculated the odds or risk ratios and their 95% confidence intervals to quantify the effects of the independent variables on the response variables. The models were built using the MASS package (www.stats.ox.ac.uk/pub/MASS4/) in R. The comparison tables were generated using the Stargazer package (Hlavac, 2018). The analysis source code, detailed quality checks and all Supplementary material are available in GitHub (https://github.com/M1gus/AirPollutionCOVID19). The analysis notebook is available at the following link: https://m1gus.github.io/AirPollutionCOVID19/. Statistical significance was defined as *p* ≤ 0.05.

## 3 Results

### 3.1 Links between regional nitrogen oxide and ozone levels and COVID-19 in England

We analysed the associations between cumulative numbers of COVID-19 cases and deaths with the concentrations of three major air pollutants recorded between 2018 and 2019, when no COVID-19 cases were reported. Due to differences in data availability for each air pollutant, we only included annual mean values of daily measurements, which was the most consistent aggregation type reported for all air pollutants described in this analysis. We started by analysing publicly available data from seven regions in England (Table 1). For each region, a minimum of 2000 SARS-CoV-2 infections and 200 deaths were reported by PHE from February 1 to April 8, 2020, which was approximately two weeks after the UK was placed into lockdown (Fig. 1).

The spatial pattern of COVID-19 deaths matched the geographical distribution of COVID-19-related cases, with the largest numbers of COVID-19 deaths occurring in London and in the Midlands (Fig. 2). According to previous studies, those two areas present the highest annual average concentration (μg/m^3^) of nitrogen oxides (Pannullo et al., 2017). In addition, ground-level ozone concentrations have been previously shown to vary significantly with latitude and altitude, depending on the concentration of ozone in the free troposphere, long-range transport and emission of its precursor (Hagenbjork et al., 2017). Therefore, we sought to determine if spatial variations in the levels of nitrogen oxides, in particular nitrogen dioxide (NO_2_) and nitrogen oxide (NO), as well as ground-level ozone concentrations in England are associated with increased numbers of COVID-19 infections and mortality. We applied a negative binomial regression model to estimate the association between each air pollutant with the cumulative number of both COVID-19 cases and deaths at the regional level (Supplementary Tables 2 and 3). The model was chosen based on the data type (count data) and log likelihood and AIC scores (Akaike et al., 1998). Population density, a confounding factor, was added to this model as an independent variable to account for differences in the number of inhabitants across regions. The levels of nitrogen oxide and nitrogen dioxide are significant predictors of COVID-19 cases (*p* < 0.05), independent of the population density (Supplementary Table 2). We next applied a similar method to assess the association with the number of COVID-19 deaths (Supplementary Table 3). Ozone, nitrogen oxide and nitrogen dioxide levels are significantly associated with COVID-19 deaths, together with the population density.

Taken together, the negative binomial regression models of both COVID-19 cases and deaths (Supplementary Tables 2 and 3) show that nitrogen dioxide, nitrogen oxide and ozone levels are significant predictors of COVID-19-related death, after accounting for the population density. This study provides the first evidence that SARS-CoV-2 cases and deaths are associated with regional variations in air pollution across England.

Nitrogen oxides are the main contributors to increased numbers of COVID-19 deaths and cases in the early phase of the pandemic.

**Fig. 3:**
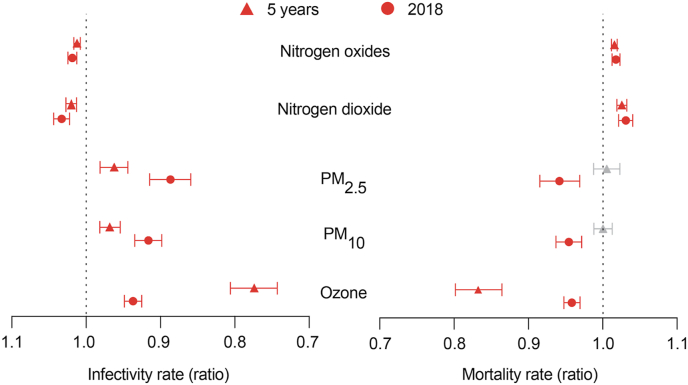
Cases and deaths in local authorities.

We next sought to increase both the resolution and accuracy of our analysis. We gathered data on COVID-related cases and deaths from all the local authorities in England and expanded the number of the pollutant species (n = 5). We also retrieved the longitude and latitude for each local authority. The levels of ozone, nitrogen oxide, nitrogen dioxide and PM with aerodynamic diameters of 2.5 and 10 μm (PM_2.5_ and PM_10_, respectively) are reported as averages of the 10 values measured nearest the centre of each local authority in England. Local authority-level population density, mean annual earnings and median age were included as potential confounding variables (Fig. 1). We calculated the estimated regression coefficients of each variable and their respective mortality and infectivity rate ratios (Fig. 3 and Supplementary Tables 4 and 5) relative to the different air pollutants mentioned. In our single-year model (2018), higher nitrogen dioxide levels predict an increase in COVID-19 deaths and cases in the early phase of the pandemic (Fig. 3). Moreover, the levels of nitrogen dioxide have a infectivity rate ratio of 1.033 [95% confidence interval (CI): 1.043–1.022] and mortality rate ratio of 1.031 [95% CI: 1.040–1.021], indicating that a 1 μg/m^3^ increase in nitrogen dioxide concentration in 2018 was associated with 3.3% more cases and 3.1% more deaths in England. Similar to nitrogen dioxide, the levels of nitrogen oxides show mortality and infectivity rate ratios of approximately 1.01 (Fig. 3). The incidence rate ratios of cases and deaths for ozone levels are less than 1, indicating that higher ozone levels lead to lower numbers of deaths and cases. PM_2.5_ and PM_10_ are negatively associated with the number of cases, and they are not significant predictors of the number of COVID-19-related deaths based on 2018 air pollution data. To determine the effect of spatial-temporal variations in air pollution exposure in England, we further increased the temporal scale of our analysis to include temporally averaged air pollution data for the years 2014–2018 (Fig. 3). Our results show that the estimated effect of air pollution on COVID-19 mortality and infectivity remains roughly constant over the multi-year modelling period (Fig. 3). Levels of nitrogen oxides and nitrogen dioxide remain significantly associated with an increase in COVID-19 infectivity [OR: 1.012 95% CI: 1.016–1.008 and OR: 1.020 95% CI: 1.027–1.013, respectively] and mortality [OR: 1.015 95% CI: 1.019–1.011 and OR: 1.025 95% CI: 1.032–1.019, respectively] approximately one month after England was placed on lockdown (Fig. 3). Similarly, we found that an increase in long-term average of ozone is negatively associated with both COVID-19 mortality [OR: 0.832 95% CI: 0.864–0.801] and infectivity [OR: 0.774 95% CI: 0.806–0.743]. In the case of PM_2.5_ and PM_10_, we found a negative and statistically significant association between the long-term average of these air pollutants and COVID-19 cases [OR: 0.962 95% CI: 0.981–0.944 and OR: 0.968 95% CI: 0.981–0.955, respectively] but not for deaths.

Levels of PM pollutants and nitrogen oxides are associated with an increase in SARS-CoV2 infections in UK Biobank participants living in England.

**Fig. 4:**
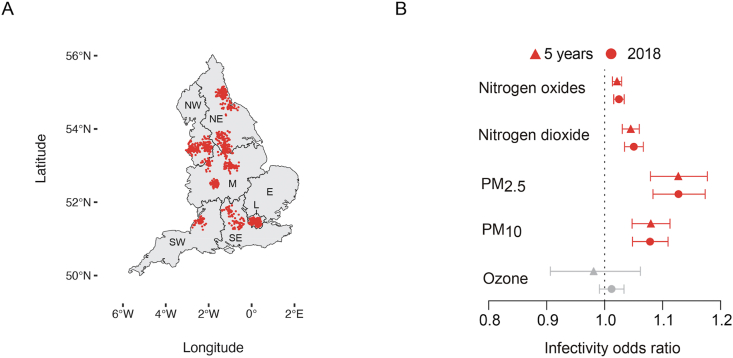
Distribution and infectivity data from the UK Biobank. A) Distribution of UK Biobank subjects included in the current analysis. B) Odds ratios and respective 95% CIs for the relationship between individual exposure to several air pollutants and the number of lab-confirmed COVID-19 cases. Triangles refer to the results obtained when the long-term average (five years, 2014–2018) in the concentration of each air pollutant was taken into account and circles refer to the results obtained when the primary measure of exposure was air pollution levels in 2018. Red indicates significant associations (*p* ≤ 0.05), while grey indicates a lack of significance (*p* > 0.05).

We next used information from the UK Biobank to further assess whether people exposed to increased pollution levels are more likely to contract SARS-CoV-2 at the individual scale. This resource contains data from more than half a million UK volunteers recorded across multiple years. We obtained COVID-19 data reported by the UK Biobank up to and including April 26, 2020. This dataset contained COVID-19 test results for 1464 participants, of whom 664 were diagnosed as positive for COVID-19. The location of each subject included in the analysis is shown in Fig. 4A. Compared to the local authority case model, the UK Biobank analysis provides a higher resolution air pollution estimate (less than 2 km away from their self-reported address) and includes potentially asymptomatic cases.

In our model, we accounted for a list of confounding variables (Supplementary Table 1), which we selected based on a previous study (Williamson et al., 2020). Our analysis identified PM_2.5_ and PM_10_ as significant predictors of increased SARS-CoV-2 infectivity based on our single-year exposure model (Fig. 4B). The odds ratios are 1.127 [CI: 1.173–1.083] and 1.078 [CI: 1.109–1.048] for PM_2.5_ and PM_10_, respectively (Fig. 4B). When the long-term averages of PM_2.5_ and PM_10_ levels were considered, the estimated coefficients remain positive and statistically significant, with a similar magnitude to those identified based on 2018 air pollution data alone (Fig. 4B). That is, we found that a single unit increase in PM_2.5_ levels was associated with a statistically significant 12% increase in COVID-19 cases, regardless of the primary exposure measure (i.e., single year or multiyear exposure). For PM_10_, a one-unit increase was associated with approximately 8% more COVID-19 cases in the UK biobank. Interestingly, these results are inconsistent with data obtained from the subregional models, where PM was not found to predict the number of cases, which may be related to the lack of individual-level data. Nonetheless, both our subregional and individual-level models suggest that the levels of nitrogen oxides and dioxide were positively associated with COVID-19 infectivity, with an odds ratio of approximately 1.03 for both the single-year and multi-year model (Fig. 4B). Based on our results, we predict that an increase of only 1m3 in the long-term average of nitrogen dioxide levels increased COVID-19 cases by 4.5% [95% CI: 5.99%–3.05%] while a similar increase in nitrogen oxides was associated with approximately 2% more cases [95% CI: 2.92%–1.35%]. Conversely, ozone levels are not significant predictors of infectivity at the individual level, although they were significantly associated with deaths and cases at the subregional level (Fig. 3, Fig. 4B). In addition to air pollution, we observed an association between current smokers and a lower likelihood of COVID-19 positivity than previous and non-smokers. However, according to our model, population density and predisposing health factors, such as age, sex, diabetes and a previous history of cancer and lung problems, are not predictors of the probability of being infected (Supplementary Table 6).

## 4 Discussion

Here, we identified associations between air pollution and COVID-19 deaths and cases in England, expanding on previous evidence linking high mortality rates in Europe with increased toxic exposure to air pollutants (Conticini et al., 2020; Ogen, 2020). Air pollution exposure and health impact estimates have been suggested to mainly depend on the resolution at which they are evaluated (Stroh et al., 2007). Therefore, we first calculated the effects of air pollution on COVID-19 mortality and spread using regional, coarse resolution data, and then high-resolution, individual-level observations obtained from the UK Biobank. By employing finer resolution grids, we found statistically significant evidence that an increase in the long-term average of PM_2.5_ is associated with the largest increase in COVID-19 infectivity in England.

According to our initial findings, regional variations in nitrogen oxide and ozone concentrations in England predict the numbers of COVID-19 cases and deaths, independent of the population density. However, overall uncertainties for modelled exposure estimates at the regional scale (Stroh et al., 2007) led us to obtain increased spatial resolution. Using highly granular local authority-level measurements, we found that a 1 μg/m^3^ increase in the long-term average of nitrogen oxides and dioxide levels was associated with a 1.5% and 2.5% increase in COVID-19 related mortality, respectively. Notably, these findings are consistent with studies conducted during the previous SARS outbreak, where long-term exposure to air pollutants predicted adverse outcomes in patients infected with SARS in China (Cui et al., 2003). Although nitrogen oxides are key ozone precursors, the relationship between these gases and ozone is nonlinear in ozone chemistry (Kelly and Gunst, 1990). Therefore, the negative associations between ozone levels and COVID-19 infection and mortality may be attributed to reduced nitrogen oxide conversion to ozone in urban areas, a phenomenon previously reported for areas with heavy traffic (Hagenbjork et al., 2017; Melkonyan and Kuttler, 2012). In addition, given the highly reactive nature of ozone, the inverse relationship between ozone levels and COVID-19 is consistent with increased nitric oxide scavenging close to points of emissions (Lefohn et al., 2010).

Although the molecular mechanisms underlying the relationship between pollutant exposure and COVID-19 remain to be determined experimentally, they are hypothesised to include the stimulation of chronic, background pulmonary inflammation (Ogen, 2020). Chamber studies have shown that ambient nitrogen dioxide induces infiltration of the airways by inflammatory cells in healthy volunteers (Ghio et al., 2000; Sandström et al., 1989 (Sandström et al., 1991),). In addition, exposure to these pollutants may inhibit pulmonary antimicrobial responses, reducing clearance of the virus from the lungs and promoting infectivity. Reduced phagocytic function is well documented after the exposure of macrophages to PM (Becker et al., 2003; Lundborg et al., 2006; Selley et al., 2020) and is suggested to be the mechanism that enhances viral infection in mice exposed to nitrogen dioxide (Rose et al., 1988). Acute exposure to nitrogen oxides has also been shown to decrease pulmonary function by inducing systemic oxidative stress (Guarnieri and Balmes, 2014). Both the MESA-Air and Framingham cohorts demonstrated that long-term exposure to air pollution is linked to chronic reductions in endothelial function (Krishnan et al., 2012; Wilker et al., 2014). Endothelial dysfunction may result in changes in arterial stiffness and afterload, which may translate into persistent hypertension. In this context, Faustini and colleagues (Faustini et al., 2014) reported that a 10 μg/m^3^ increase in the annual concentration of two traffic-related pollutants, nitrogen dioxide and PM_2.5_, is associated with large increases in both respiratory and cardiovascular mortality. As respiratory and cardiovascular diseases represent potential risk factors for COVID-19 related mortality, these studies support the hypothesis that long-term exposure to several air pollutants enhances the risk of severe COVID-19 outcomes by weakening the respiratory, cardiovascular and immune systems, thus facilitating viral invasion and severe outcomes (Conticini et al., 2020; Kulkarni et al., 2020).

Using individual-level data, our UK Biobank model indicated that exposure to PM_2.5_ and PM_10_ increases the risk of COVID-19 infection, in addition to nitrogen oxides, which were previously identified as major contributors to COVID-19 infectivity in the regional and subregional analysis. The observation that exposure to PM_2.5_ and PM_10_ increases the risk of COVID-19 infection conforms to the hypothesis that viruses attach to air pollutants (Reche et al., 2018), potentially explaining the propagation of SARS-CoV-2 and its infectious capacity. Nonetheless, the results of our individual-level analysis are inconsistent with our local authority models, where PM_2.5_ and PM_10_ were found to be negatively associated with the infectivity rate. In this context, it must be emphasised that the ecological design of our subregional analysis likely led to some degree of exposure misclassification. Previous studies have shown that the temporal and spatial scales of exposure assessment may influence the magnitude of reported associations between air pollutant exposure and mortality (Crouse et al., 2020). For instance, Crouse and colleagues (Crouse et al., 2020) observed that the magnitude of association between PM_2.5_ and mortality in the US is sensitive to the spatial scale of the assessment, with stronger associations found at smaller spatial scales particularly for respiratory and lung cancer mortality. In support of our individual-level findings, Cole and colleagues (Cole et al., 2020) recently used a similar approach to investigate the relationship between air pollution and COVID-19 using subregional data from over 355 municipalities in the Netherlands. Their analysis showed that, after accounting for a wide range of confounders, including socioeconomic and sociodemographic factors, a one unit increase in PM_2.5_ concentrations is associated with 9.4 more Covid-19 cases.

In addition, our findings are comparable to the observations of Wu et al. (2020) in the US and another study from Northern Europe where levels of PM_2.5_ were found to be strongly associated with COVID-19 incidence, after adjusting for multiple demographic and health-related confounders (Andree, 2020). However, this report is the first study to employ individual-level data to assess the relationship between air pollution exposure and COVID-19, after controlling for individual characteristics such as age and underlying health conditions obtained by the UK Biobank. By modelling air quality estimates based on the nearest available measurements to individuals’ residences, our modelling strategy helped to minimise the potential for ecological bias and exposure misclassification errors (Miller et al., 2007). Furthermore, although considerable anecdotal evidence suggests that air quality is associated with COVID-19 outcome (Conticini et al., 2020; Ogen, 2020; Wu et al., 2020), most studies to date have been unable to accurately quantify the number of COVID-19 cases due to limited testing capacity. In England, government guidelines have prioritised testing for symptomatic COVID-19 patients, meaning that official figures do not include the growing number of people who are asymptomatic or are self-isolating at home due to mild COVID-19 symptoms. In contrast, all UK Biobank participants included in this study were subjected to COVID-19 testing since the beginning of the pandemic. Because a large proportion of COVID-19 infections are asymptomatic (Day, 2020; Nishiura et al., 2020), the UK Biobank model provides greater sensitivity to the analysis of infection rates compared to ecological models. We suggest that these differences may partly explain the conflicting results for PM_2.5_ and PM_10_ observed between our subregional and individual-level models.

Despite some notable advantages in inferring the relationship between COVID-19 infectivity and air quality, it is important to acknowledge that our individual-level analysis presented some limitations. First, our assessment of exposure remains inherently limited because the degree to which ambient monitoring stations represent the exposure of the subject is imperfect. For instance, we were unable to assess microclimate differences in exposure or details regarding the subjects’ activity and location, such as the time spent in traffic and indoors. Therefore, questions remain concerning the generalisability of the above findings, as microenvironmental (e.g., work, home, school, etc.) and behavioural factors (e.g., mobility) profoundly affect an individual’s exposure to air pollution (Ozkaynak et al., 2013). Though the incorporation of these factors is problematic in the midst of a pandemic, future work should address the confounding effects of additional variables to obtain more accurate PM exposure estimates (Pansini and Fornacca, 2020; Zhu et al., 2020b). Second, it has become clear over the course of the pandemic that confounding factors in addition to those considered in the current study, such as ethnicity, are also associated with COVID-19 infectivity and mortality rates (Brandt et al., 2020; Dutton, 2020). A caveat to the use of UK Biobank is the limited representation of ethnic minority groups because the large majority of the participants are of white ethnicity. A recent report by the ONS suggested that the correlation between air pollution and COVID-19 mortality in England becomes weaker once ethnicity is controlled for as a confounding variable (Dutton, 2020). This finding suggests that either air pollution leads to disproportionate outcomes in ethnic minority groups or that the estimated relationship between air pollution and COVID-19 is confounded by the strong relationship between the distribution of ethnic groups in England and highly polluted areas. Therefore, our results should be interpreted in the context of our modelling design and future studies should address the relationship between COVID-19 and air pollution after taking into account the confounding effect of ethnicity.

Our findings suggest that long-term exposure to poor AQ increases the risks of COVID-19 infection and mortality in the UK, in line with the results obtained from recent studies conducted in northern Italy (Conticini et al., 2020), Europe (Cole et al., 2020; Ogen, 2020), and the USA (Liang et al., 2020; Wu et al., 2020). Our results provide compelling evidence of a statistically significant relationship between nitrogen oxides and dioxide levels and COVID-19 mortality at the regional and subregional level. This relationship persists after controlling for individual-level characteristics and indicates that prolonged exposure to these urban traffic-related air pollutants may increase the risk of severe COVID-19 outcome. Our individual-level models further indicate that an increase of 1 m^3^ the long-term average of PM_2.5_ was associated with an increase of 12% in COVID-19 cases in England. A comparable effect was observed for PM_10_, whereby a one-unit increase was associated with an approximately 8% increase in COVID-19 cases. Future studies may expand on these observations and address additional confounders, including comorbidities, race, meteorological trends and differences between regional health regulations and their ICU capacities. In light of the evidence presented herein, we believe that air pollution factors should be considered when estimating the SARS-CoV-2 infection rate (R_0_). Our results emphasise the importance of strengthening efforts to tighten air pollution regulations for the protection of human health, both in relation to the COVID-19 pandemic and for the mitigation of potential future diseases.

This flowchart summarizes how raw data were extrapolated, processed and analysed. Blue indicates data sources, whereas red and green indicate the type of model employed and the final output, respectively. Population density data (person/km^2^) were derived from ONS and used to account for region-specific differences in population size across England; COVID-19 case and death data were obtained from PHE, NHS and ONS, respectively. Air pollution data from each monitoring station were manually curated using DEFRA’s Air Quality Spatial Object Register and aggregated into statistical regions. ONS, Office for National Statistics; PHE, Public Health England; NHS, National Health Service; EEA, European Environmental Agency.

Regional English heatmaps of reported deaths and diagnosed COVID-19 cases through April 8, 2020 (top row), as well as AQ values for the indicated pollutants (bottom row).

Summary of infectivity and mortality rate ratios and respective 95% CIs at the local authority level. Triangles refer to the results obtained when the long-term average (five years, 2014–2018) in the concentration of each air pollutant was taken into account whereas circles refer to the results obtained when the primary measure of exposure was air pollution levels in 2018. Red indicates significant associations (*p* ≤ 0.05), while grey indicates a lack of significance (*p* > 0.05). See also Supplementary Table 4 for a detailed description of the model.

## Author contribution

Marco Travaglio, planned and designed the study, collected the data, treated and analysed the data, developed the models and wrote the RMD file, wrote the manuscript with the support of, conducted this study while in self-isolation due to the current pandemic. Yizhou Yu, planned and designed the study, collected the data, treated and analysed the data, developed the models and wrote the RMD file, wrote the manuscript with the support of, conducted this study while in self-isolation due to the current pandemic. Rebeka Popovic, planned and designed the study, collected the data, provided guidance regarding study of air pollution toxicity, conducted this study while in self-isolation due to the current pandemic. Liza Selley, provided guidance regarding study of air pollution toxicity, conducted this study while in self-isolation due to the current pandemic. Nuno Santos Leal, collected the data, treated and analysed the data, provided guidance regarding study of air pollution toxicity, conducted this study while in self-isolation due to the current pandemic. Luis Miguel Martins, planned and designed the study, conducted this study while in self-isolation due to the current pandemic.

## Declaration of competing interest

The authors declare that they have no known competing financial interests or personal relationships that could have appeared to influence the work reported in this paper.
